# Prognostic impact of the AML ELN2022 risk classification in patients undergoing allogeneic stem cell transplantation

**DOI:** 10.1038/s41408-022-00764-9

**Published:** 2022-12-19

**Authors:** Madlen Jentzsch, Lara Bischof, Jule Ussmann, Donata Backhaus, Dominic Brauer, Klaus H. Metzeler, Maximilian Merz, Vladan Vucinic, Georg-Nikolaus Franke, Marco Herling, Uwe Platzbecker, Sebastian Schwind

**Affiliations:** Department for Hematology, Cell Therapy and Hemostaseology, University of Leipzig Medical Center, Leipzig, Germany

**Keywords:** Acute myeloid leukaemia, Disease-free survival, Risk factors

## Abstract

For most patients with acute myeloid leukemia (AML), an allogeneic hematopoietic stem cell transplantation (HSCT) offers the highest chance of cure. Recently, the European LeukemiaNet (ELN) published updated recommendations on the diagnosis and risk classification in AML based on genetic factors at diagnosis as well as a dynamic adjustment (reclassification) according to the measurable residual disease (MRD) status for the favorable and intermediate risk groups. Validation of the ELN2022 risk classification has not been reported. We retrospectively analyzed 522 AML patients who received an HSCT at a median age of 59 (range 16–76) years. For patients with adequate material available and in remission prior to HSCT (*n* = 229), the MRD status was evaluated. Median follow-up after HSCT was 3.0 years. ELN2022 risk at diagnosis was in 22% favorable, in 26% intermediate, and in 52% adverse. ELN2022 risk at diagnosis is associated with the cumulative incidence of relapse/progression (CIR), event-free survival (EFS), and overall survival (OS) in the whole patient cohort, as well as the subgroup of patients transplanted in first remission. However, the risk stratification based on the ELN2022 classification did not significantly improve outcome prognostication in comparison to the ELN2017 classification. In our study, the newly added group of patients with myelodysplasia-related gene mutations did not have adverse outcomes. Re-classifying these patients into the intermediate risk group and adjusting the grouping for all AML patients by MRD at HSCT, led to a refined and improved risk stratification, which should be validated in independent studies.

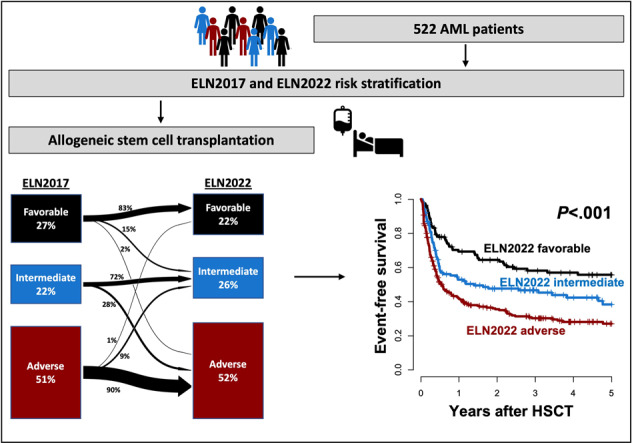

## Introduction

In 2022, an expert panel on behalf of the European LeukemiaNet (ELN) defined and published a revised risk classification system for acute myeloid leukemia (AML), which was established in 2010 and was revised initially in 2017 [1–3]. While the distribution into three genetic risk groups at diagnosis—as introduced by the ELN2017 classification - was maintained, some relevant changes were made, reflecting new insights into AML disease biology and risk stratification. Among the major changes, *CEBPA* mutations categorized as ELN2022 favorable risk are now restricted to in-frame mutations in the basic leucine zipper (bZIP) region, irrespective of them occurring as mono- or biallelic [4–6]. Moreover, the former division into high or low *FLT3*-ITD allelic ratio (AR) was abandoned, allocating all patients harboring an *FLT3*-ITD to the ELN2022 intermediate risk group, irrespective of the presence or absence of an *NPM1* mutation. *NPM1* mutations continue to indicate favorable outcomes in the absence of an *FLT3*-ITD, with the new exception of co-occurring adverse risk cytogenetics, which now indicates ELN2022 adverse risk. Also, the definition of a complex karyotype changed with the exclusion of hyperdiploid karyotypes with multiple trisomies from this group. Already in the ELN2017 risk classification, mutations in the three genes *ASXL1*, *RUNX1*, and *TP53* have been introduced as new adverse risk prognostic factors. Now additionally, so-called myelodysplasia-related gene mutations, i.e., in the genes *BCOR*, *EZH2*, *SF3B1*, *SRSF2*, *STAG2*, *U2AF1*, or *ZRSR2*, define ELN2022 adverse risk in the absence of favorable risk genetics. Finally, a 10% variant allele frequency (VAF) threshold has been introduced for *TP53* mutations to allocate individuals to ELN2022 adverse risk [3].

Previous work has shown the prognostic power of the ELN risk classifications published in 2010 [7, 8] and 2017 [9–12]. While this was seen irrespective of whether the patients were consolidated by chemotherapy or allogeneic hematopoietic stem cell transplantation (HSCT) [7, 9], the separation of outcome curves according to the ELN risk groups seemed to be less pronounced in individuals that received an allogeneic HSCT, thereby strengthening the use of HSCT consolidation in higher-risk AML patients. In contrast to previous ELN risk stratification systems, the feasibility of the latest update published very recently in 2022, remains to be demonstrated, which was the main objective of our study.

## Patients and methods

### Patients and treatment

We retrospectively analyzed 522 AML patients (median age 59, range 16–76 years) who received an allogeneic HSCT between January 2000 and December 2021 at our center and who had adequate diagnostic information available to group them unambiguously into one of the three ELN2022 risk groups. Patients were either treated with standard intensive cytarabine-based chemotherapy or received hypomethylating agents with or without venetoclax as induction treatment. Remissions status at HSCT was either first CR or first CR with incomplete peripheral cell count recovery (CRi, *n* = 337, 65%), later CR/CRi (*n* = 72, 14%), or relapsed/refractory disease (*n* = 113, 22%). Patients received either non-myeloablative (50%), reduced intensity (23%) [13–15], or myeloablative conditioning (27%). Details on the applied induction therapy, conditioning regimens, and immunosuppression are given in the Supplementary Information. Further patients’ characteristics at diagnosis and HSCT are given in Table 1 and Supplementary Table S1. Median follow-up after HSCT for patients alive was 3.0 years. Written informed consent was obtained in accordance with the Declaration of Helsinki. Data analyses were approved by the Institutional Review Board of the University Hospital Leipzig.

**Table 1:** Patients’ characteristics according to the ELN2022 genetic risk at diagnosis (*n* = 522).

	All patients *n* = 522	ELN22 favorable *n* = 114	ELN22 intermediate *n* = 137	ELN22 adverse *n* = 271	*P*
Sex, *n* (%)					0.24
male	261	54 (47)	62 (45)	145 (54)	
female	261	60 (53)	75 (55)	126 (46)	
Disease origin, *n* (%)					<0.001
secondary/treatment related	177	19 (17)	26 (19)	132 (49)	
de novo	345	95 (83)	111 (81)	139 (51)	
Hemoglobin, g/dL					0.13
median (range)	8.5 (3.4–14.9)	8.5 (3.8–14.7)	9 (3.4–5.6)	8.4 (4.2–14.9)	
Platelet count, x 10^9^/L					0.08
median (range)	60 (1–950)	80 (3–442)	63 (7–289)	49 (1–950)	
WBC, x 10^9^/L					<0.001
median (range)	6.4 (0.1–385)	18.6 (1–366)	13.6 (0.6–385)	3.6 (0.1–238)	
Blood blasts, %					<0.001
median (range)	23 (0–97)	32 (0–97)	42 (0–97)	14 (0–88)	
Bone marrow blasts, %					<0.001
median (range)	50 (0–99)	50 (14–100)	75 (21–99)	40 (0–95)	
CD34+/CD38- cell burden					<0.001
median (range)	0.7 (0–75)	0.1 (0–20.6)	0.5 (0–32.5)	2.1 (0–75)	
LDH, ukat/l					0.34
median (range)	6.12 (1.4–30.0)	7.1 (2.9–30.0)	8.6 (2.5–30.0)	5.2 (1.4–30.0)	
RDW, %					0.02
median (range)	16.1 (12.2–29.6)	16.1 (13.3–24)	16.2 (12.4–25.2)	17.1 (12.2–29.6)	
Normal karyotype, *n* (%)					<0.001
absent	182	48 (44)	48 (36)	233 (86)	
present	329	60 (56)	85 (64)	37 (14)	
*DNMT3A* mutation, *n* (%)					0.002
wild type	200	44 (65)	54 (67)	102 (84)	
mutated	70	24 (35)	27 (33)	19 (16)	
*FLT3*-TKD mutation, *n* (%)					0.007
wild type	391	82 (82)	107 (88)	202 (94)	
mutated	47	18 (18)	15 (12)	14 (6)	
*KIT* mutation, *n* (%)					0.004
wild type	183	43 (86)	49 (100)	91 (97)	
mutated	10	7 (14)	0 (0)	3 (3)	
*JAK2* mutation, *n* (%)					0.04
wild type	144	29 (100)	39 (93)	76 (85)	
mutated	16	0 (0)	3 (7)	13 (15)	
*TET2* mutation, *n* (%)					<0.001
wild type	143	21 (75)	48 (98)	74 (75)	
mutated	33	7 (25)	1 (2)	25 (25)	
*WT1* mutation, *n* (%)					0.02
wild type	124	23 (96)	33 (87)	68 (99)	
mutated	7	1 (4)	5 (13)	1 (1)	
Age at HSCT, years					<0.001
median (range)	59.1 (16.3–76.4)	56.3 (16.3–76.4)	56.3 (19.4–75.9)	61.5 (20.3–76.2)	
Remission status at HSCT, *n* (%)					<0.001
CR/CRi	409	106 (93)	118 (86)	185 (68)	MRD^pos^ vs MRD^neg^
CR/CRi, MRD^neg^	166	35 (33)	39 (28)	53 (20)	
CR/CRi, MRD^pos^	102	34 (32)	34 (25)	34 (13)	0.40
CR/CRi, no MRD information	141	37 (35)	45 (33)	98 (36)	
CR/CRi1	337	81 (76)	90 (76)	166 (90)	CR/CRi1 vs CR/CRi2 0.003
CR/CRi2	70	24 (23)	27 (23)	19 (10)	
CR/CRi3	2	1 (1)	1 (81)	0 (0)	
PR/relapsed/refractory	113	8 (7)	19 (14)	86 (32)	
Conditioning regimen, *n* (%)					
NMA	261	59 (52)	65 (47)	137 (51)	0.77
RIC	120	12 (11)	26 (19)	82 (30)	<0.001
MAC	141	43 (38)	46 (34)	52 (19)	<0.001
HCT-CI Score, *n* (%)					
0	203	63 (58)	43 (33)	97 (39)	<0.001
1/2	157	26 (24)	52 (40)	79 (32)	0.03
≥ 3	134	20 (18)	35 (27)	74 (30)	0.08
Donor type, *n* (%)					
matched related	88	28 (25)	23 (17)	37 (14)	0.05
unrelated, HLA matched	334	67 (59)	90 (66)	177 (66)	0.41
HLA mismatched	96	17 (15)	19 (14)	50 (19)	0.46
haploidentical	13	2 (2)	5 (4)	6 (2)	0.66
Donor sex, *n* (%)					0.49
female into male	69	19 (17)	17 (13)	33 (12)	
all others	445	93 (83)	119 (88)	233 (88)	
CMV status, *n* (%)					0.14
recipient+/ donor –	187	42 (38)	38 (28)	99 (37)	
all others	339	70 (63)	99 (72)	170 (63)	
Acute GvHD ≥ grade 2, *n* (%)					0.36
absent	340	71 (73)	84 (71)	184 (78)	
present	113	26 (27)	34 (29)	53 (22)	
Chronic GvHD, *n* (%)					
absent	165	36 (43)	41 (44)	88 (53)	0.20
limited	50	13 (16)	21 (22)	16 (10)	<0.001
extended	127	34 (41)	32 (34)	61 (37)	0.65

*CMV* cytomegalovirus, *CR* complete remission, *CRi* complete remission with incomplete peripheral recovery, *DNMT3A* DNA methyltransferase 3 alpha gene, *ELN* European LeukemiaNet, *FLT3* fms-like tyrosine kinase, *FLT3-ITD* internal tandem duplication of the FLT3 gene, *FLT3-TKD* tyrosine kinase mutations in the FLT3 gene, *GvHD* graft-versus-host disease, *Hb* hemoglobin, *HLA* human leukocyte antigen, *HCT-CI* hematopietic cell transplantation comorbidity index, *HSCT* hematopoietic stem cell transplantation, *JAK2* Janus kinase 2 gene, *LDH* lactate dehydrogenase, *MRD* measurable residual disease, *PB* peripheral blood, *PR* partial remission, *RDW* red cell distribution width, *TET2* Tet methylcytosine dioxygenase 2 gene, *WBC* white blood count, *WT1* Wilm’s tumor gene 1.

### Cytogenetics and molecular markers

Cytogenetic analyses at diagnosis were performed using standard techniques of banding and in situ hybridization. Pretreatment genomic DNA was screened for the presence of *FLT3*-ITD, as well as the mutation status of the genes *CEBPA* and *NPM1* as previously described [9, 16]. In patients with adequate samples available, the diagnostic mutation status of 54 genes recurrently mutated in myeloid malignancies was evaluated using next-generation sequencing (Illumina, San Diego, CA, USA) as previously described [17]. *ASXL1* mutations at codon 646 were validated by applying a proofreading polymerase-based Sanger sequencing approach [17].

### MRD assessment prior to allogeneic HSCT

For patients transplanted in CR or CRi with adequate bone marrow or peripheral blood material acquired ≤28 days prior to HSCT available (*n* = 229), the MRD status was assessed using digital droplet polymerase chain reaction (PCR) for at least one of the targets *NPM1* mutation, *BAALC/ABL1* copy numbers and *MN1/ABL1* copy numbers or using quantitative reverse transcriptase PCR for *WT1/ABL1* expression levels adapting the previously published cut-offs [18–21]. Patients with at least one positive test result were regarded as pre-HSCT MRD-positive.

### Statistical analyses

Using the Fine and Gray method, cumulative incidence of relapse (CIR) was calculated from HSCT to relapse considering its competing risk non-relapse mortality (NRM), which was calculated from HSCT to death without relapse [22]. Event-free survival (EFS) and overall survival (OS) were calculated from HSCT until death from any cause and relapse or death, respectively, using the Kaplan–Meier method and groups were compared using the log-rank test. For outcome calculations at 3 years after HSCT, the respective 95% confidence intervals (CI) are presented in Supplementary Table S2. Associations with baseline clinical, demographic, and molecular features were compared using the Kruskal–Wallis test and Fisher’s exact tests for continuous and categorical variables, respectively. Receiver operating characteristic (ROC) curves were used as graphical plots to depict the predictive value of selected variables. All *P* values are two-sided. All statistical analyses were performed using the R statistical software platform (version 4.0.2) [23].

## Results

### Comparison of the ELN2017 and ELN2022 genetic risk classifications

**Fig. 1:**
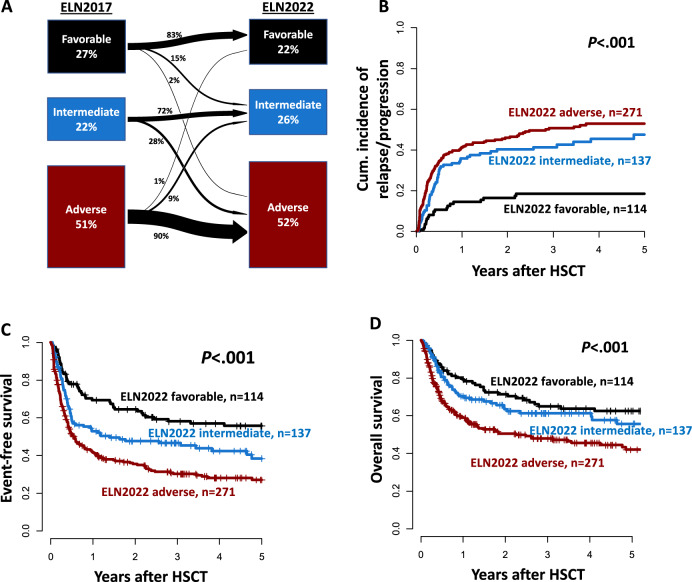
Risk distribution and outcomes according to the ELN2022 genetic risk groups at diagnosis. **A** Transition plot of risk distribution between the ELN2017 and ELN2022 risk stratification systems at diagnosis. **B** Cumulative incidence of relapse, **C** Event-free survival, and **D** Overall survival according to the ELN2022 genetic risk groups in the whole patient cohort (*n* = 522).

Of the analyzed AML patients, 114 (22%) were classified to have favorable, 137 (26%) to have intermediate, and 271 (52%) to have adverse ELN2022 risk at diagnosis. Comparing the ELN2017 and the new ELN2022, 83% of favorable, 72% of intermediate, and 90% of adverse risk patients kept their allocation, while the risk of the remaining patients changed as depicted in Fig. 1A. When comparing the c-statistic (area under the curves [AUC]) derived from the diagnostic ELN2017 and ELN2022 classification for relapse (AUC_ELN2017_ = 0.65 vs AUC_ELN2022_ = 0.61, *P* = 0.02), EFS (AUC_ELN2017_ = 0.64 vs AUC_ELN2022_ = 0.61, *P* = 0.03), and OS (AUC_ELN2017_ = 0.65 vs AUC_ELN2022_ = 0.59, *P* = 0.02) within 1 year after HSCT, the ELN2017 performed significantly better than the ELN2022 (Supplementary Fig. S1).

### Clinical and biologic characteristics within the three ELN2022 risk groups

Within the ELN2022 adverse risk group, patients were older (*P* < 0.001, Table 1), more often had secondary or treatment-related AML (*P* < 0.001), had a lower white blood cell count (*P* < 0.001), and lower blast percentages in the blood (*P* < 0.001) and bone marrow (*P* < 0.001) than patients with ELN2022 favorable or intermediate risks. While patients with ELN2022 adverse risk also less often received their allogeneic HSCT in CR/CRi (*P* < 0.001) and—of the patients transplanted in CR/CRi—less often in second than in first CR/CRi (*P* = 0.003), the MRD status at HSCT did not differ between the three ELN2022 risk groups (*P* = 0.40). Gene mutations not included in the ELN2022 risk classification differed significantly between the three risk groups: *FLT3*-TKD, *DNMT3A*, and *KIT* mutations were most frequently observed in ELN2022 favorable risk patients (*P* = 0.007, *P* = 0.002, and *P* = 0.005, respectively), *WT1* mutations most frequently in ELN2022 intermediate risk patients (*P* = 0.02), *JAK2* mutations most frequently in ELN2022 adverse risk (*P* = 0.04), and *TET2* mutations less frequently in ELN2022 intermediate risk patients (*P* < 0.001). The immunophenotype significantly differed between the three ELN2022 risk groups, including a stepwise higher diagnostic burden of the immature CD34+/CD38− cell population with higher ELN2022 risk (*P* < 0.001, for details, please see Supplementary Information).

### Prognostic relevance of the three ELN2022 risk groups

**Fig. 2:**
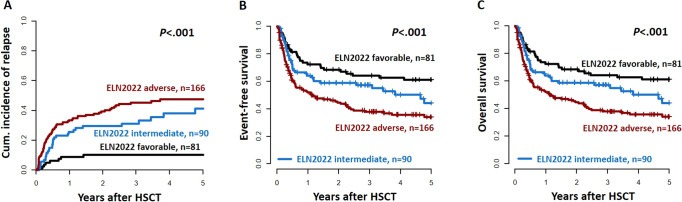
Outcomes according to the ELN2022 genetic risk groups at diagnosis within patients transplanted in first morphologic remission (*n* = 337). **A** Cumulative incidence of relapse, **B** Event-free survival, and **C** Overall survival.

In AML patients receiving an allogeneic HSCT, the allocation of patients into the three ELN2022 risk groups resulted in a significantly distinct CIR (*P* < 0.001), EFS (*P* < 0.001), and OS (*P* < 0.001, Fig. 1B–D). Three years after HSCT, in patients with ELN2022 favorable, intermediate, and adverse risk, CIR was 18, 41, and 51%, respectively; EFS was 58, 47, and 30%, respectively; and OS was 65, 61, and 48%, respectively (Supplementary Table S2). Also, in multivariate analyses, the ELN2022 risk at diagnosis remained a prognostic factor for all analyzed endpoints (Table 2). Similar results regarding the prognostic relevance of the ELN2022 risk stratification were observed when restricting the analysis to patients transplanted in morphologic remission (Supplementary Fig. S2). The best outcome separation by the ELN2022 classification was observed for patients transplanted in the first CR/CRi (Fig. 2). In contrast—although limited by lower patient numbers—in patients transplanted in the second or without CR/CRi, only patients with favorable ELN2022 risk performed better than those with intermediate or adverse risks, and no distinct outcomes were observed between the latter two groups (Supplementary Fig. S3). The ELN2022 also distinguished outcomes among patients younger (CIR *P* < 0.001, EFS *P* < 0.001, OS *P* < 0.001, Supplementary Fig. S4) and older (CIR *P* = 0.002, EFS *P* = 0.01, OS *P* = 0.10) than 60 years at HSCT, although outcome differences—especially between ELN2022 favorable and intermediate risks - were less pronounced in older AML patients.

**Table 2:** Multivariate analysis.

	Cumulative incidence of relapse		Event-free survival		Overall survival	
	HR*	*P*	HR*	*P*	HR* (95% CI)	*P*
ELN2022 risk (adverse vs intermediate vs favorable)	1.91 (1.45–2.52)	<0.001	1.59 (1.27–1.97)	<0.001	1.32 (1.03–1.70)	0.03
MRD status at HSCT (MRD^pos^ vs MRD^neg^)	3.81 (2.42–6.00)	<0.001	2.43 (1.70–3.46)	<0.001	1.70 (1.13–2.56)	0.01
Conditioning intensity (MAC vs RIC/NMA)	-	-	-	-	0.56 (0.38–0.86)	0.007
Number of remission at HSCT (no remission vs second vs first)	2.48 (1.63–3.78)	<0.001	1.60 (1.10–2.34)	0.01	-	

Variables considered in the models were those significant at α = 0.10 in univariate analyses.

For CIR endpoint, variables considered were: ELN2022 risk group, conditioning regimen (RIC/NMA vs MAC), pre-HSCT MRD status (MRD^pos^ vs MRD^neg^), and the number of remission at HSCT (no remission vs second vs first). For the EFS endpoint, variables considered were: age at HSCT (≥60 vs <60 years), ELN2022 genetic risk group, disease origin (de novo vs secondary/treatment-related), pre-HSCT MRD status (MRD^pos^ vs MRD^neg^), conditioning regimen (RIC/NMA vs MAC), and the number of remission at HSCT (no remission vs second vs first). For the OS endpoint, variables considered were: age at HSCT (≥60 vs <60 years), disease origin (de novo vs secondary/treatment-related), ELN2022 genetic risk group, pre-HSCT MRD status (MRD^pos^ vs MRD^neg^), conditioning regimen (RIC/NMA vs MAC), and the number of remission at HSCT (no remission vs second vs first).

*CI* confidence interval, *ELN2022* European LeukemiaNet 2022, *HSCT* hematopoietic stem cell transplantation, *MAC* myeloablative conditioning, *MRD* measurable residual disease, *NMA* non-myeloablative conditioning, *RIC* reduced-intensity conditioning.

*HR hazard ratio, <1 (>1) indicates a lower (higher) risk of relapse for the first category listed for the dichotomous variables for the lower (higher) values of the continuous variables.

The diagnostic qualifiers now introduced into the ELN2022 classification are discussed, and their impact on outcomes is shown in Supplementary Information and Supplementary Fig. S5.

### Outcomes according to different genetic characteristics within the three ELN2022 risk groups

**Fig. 3:**
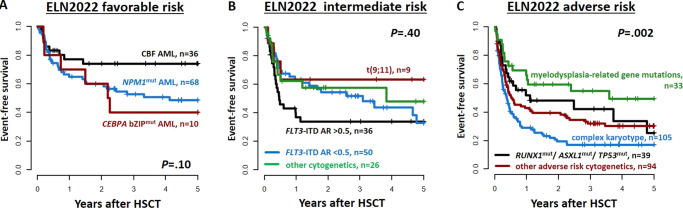
Event-free survival within the three ELN2022 risk groups at diagnosis according to the different genetic characteristics. **A** In favorable ELN2022 risk AML patients (*n* = 114), **B** in intermediate ELN2022 risk AML patients (*n* = 121), and **C** in adverse ELN2022 risk AML patients (*n* = 271). Note: *P* values for subgroup comparisons: ELN2022 favorable risk: CBF *vs NPM1*^mut^ *P* = 0.05, CBF vs *CEBPA* bZIP^mut^ *P* = 0.09, *NPM1*^mut^ vs *CEBPA* bZIP^mut^ *P* = 0.70; ELN2022 intermediate risk: *FLT3*-ITD AR <0.5 vs *FLT3*-ITD AR >0.5 *P* = 0.30, *FLT3*-ITD AR >0.5 vs *t*(9;11) *P* = 0.20, *FLT3*-ITD AR >0.5 vs other cytogenetics *P* = 0.20, *FLT3*-ITD AR <0.5 vs *t*(9;11) *P* = 0.30, *FLT3*-ITD AR <0.5 vs other cytogenetics *P* = 0.70, *t*(9;11) vs other cytogenetics *P* = 0.50). ELN2022 adverse risk: ELN2017 defined high-risk mutations vs myelodysplasia-related gene mutation *P* = 0.30, ELN2017 defined high-risk mutations (i.e., mutations in the genes: *ASXL1*, *RUNX1*, and *TP53*) vs complex karyotype *P* = 0.02, ELN2017 defined high-risk mutations vs other adverse risk karyotypes *P* = 0.40, myelodysplasia-related gene mutation vs complex karyotype *P* < 0.001, myelodysplasia-related gene mutation vs other adverse risk karyotypes *P* = 0.09, complex karyotype vs other adverse risk karyotypes *P* = 0.04.

To gain further insight into the prognostic significance of included genetic aberrations, the three ELN2022 risk groups were analyzed separately. Within the ELN2022 favorable risk group, patients with core-binding factor (CBF) AML tended to have longer EFS than patients with in-frame *CEBPA* bZIP or *NPM1* mutations (Fig. 3A), but did not differ in CIR or OS (Supplementary Fig. S6A, B).

Also, within the ELN2022 intermediate risk group, there were no significant outcome differences between AML patients with a high or low *FLT3-*ITD allelic ratio (0.5 cut-off), *t*(9;11), or other ELN2022 intermediate risk aberrations (Fig. 3B and Supplementary Fig. S6C, D).

In contrast, in patients within the ELN2022 adverse risk group, outcomes significantly differed according to the presence of a complex karyotype (at 3 years: CIR 59%, EFS 17%, OS 30%), other adverse cytogenetic aberrations (at 3 years: CIR 50%, EFS 32%, OS 54%), adverse risk gene mutation (i.e.*, ASXL1*, *RUNX1*, or *TP53*, at 3 years: CIR 47%, EFS 42%, OS 58%), or myelodysplasia-related gene mutations (at 3 years: CIR 30%, EFS 55%, OS 73%, Fig. 3C, Supplementary Fig. S6E, F, and Supplementary Table S2).

### Adjusted risk stratification according to the MRD status at HSCT

**Fig. 4:**
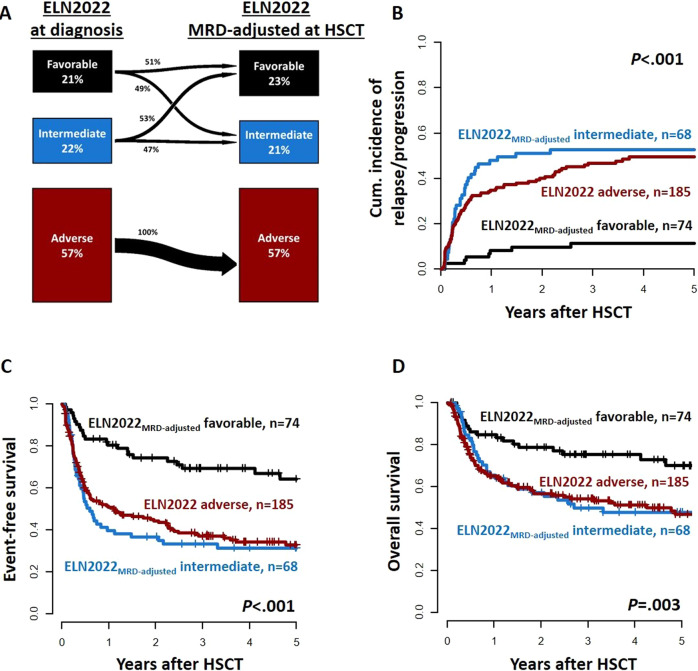
Outcomes according to the MRD-adjusted ELN2022 risk groups (favorable: ELN2022 favorable or intermediate + MRD^neg^ at HSCT; intermediate: ELN2022 favorable or intermediate + MRD^pos^ at HSCT, adverse: ELN2022 adverse). **A** Transition plot for patients’ distribution from diagnosis to HSCT, **B** Cumulative incidence of relapse, **C** Event-free survival, and **D** Overall survival in patients transplanted in CR/CRi (*n* = 327).

The ELN2022 proposed a risk adjustment (reclassification) for patients with favorable or intermediate risks at diagnosis according to the MRD status at informative time points [3]. When we adjusted the favorable and intermediate ELN2022 risk at diagnosis (${{{\mathrm{ELN2022}}}}_{{{{\mathrm{at}}}}\;{{{\mathrm{diagnosis}}}}}$) patients according to the MRD status at HSCT (${{{\mathrm{ELN2022}}}}_{{{{\mathrm{MRD - adjusted}}}}}$), 49% of favorable risk patients at diagnosis had persisting MRD and were reclassified to have intermediate risk at HSCT, while 53% of intermediate risk patients at diagnosis were MRD-negative and reclassified to have favorable risk at HSCT. Outcomes of ${{{\mathrm{ELN2022}}}}_{{{{\mathrm{MRD - adjusted}}}}}$ favorable risk patients at HSCT improved compared to the ${{{\mathrm{ELN2022}}}}_{{{{\mathrm{at}}}}\;{{{\mathrm{diagnosis}}}}}$ (at 3 years: CIR 12%, EFS 69%, OS 75%), while those of ${{{\mathrm{ELN2022}}}}_{{{{\mathrm{MRD - adjusted}}}}}$ intermediate risk patients at HSCT (at 3 years: CIR 53%, EFS 33%, OS 50%) were similar to that of adverse risk patients transplanted in morphologic CR/CRi (at 3 years: CIR 47%, EFS 37%, OS 48%, Fig. 4 and Supplementary Table S2).

### Refinement of the ELN2022 risk classification

Due to the observed ability of the ELN2022 risk classification to discriminate outcomes compared to the ELN2017 classification and the differences in outcomes within the ${{{\mathrm{ELN2022}}}}_{{{{\mathrm{at}}}}\;{{{\mathrm{diagnosis}}}}}$ adverse risk group in the transplant setting, we sought to refine it by introducing two changes. First, since we observed that patients with myelodysplasia-related gene mutations did not have adverse outcomes, we reclassified these individuals as ${{{\mathrm{ELN2022}}}}_{{{{\mathrm{at}}}}\;{{{\mathrm{diagnosis}}}}}^{{{{\mathrm{refined}}}}}$ intermediate risk at diagnosis.

Second, since the MRD status at HSCT was able to refine outcomes in all three ELN2022 risk groups, we expanded the proposed MRD-adjusted reclassification of the favorable and intermediate groups to all three risk groups. This led us to divide the patient set into three MRD-adjusted risk groups (see also Supplementary Fig. S7): The most favorable outcomes had ${{{\mathrm{ELN2022}}}}_{{{{\mathrm{at}}}}\;{{{\mathrm{diagnosis}}}}}^{{{{\mathrm{refined}}}}}$ favorable and intermediate risk patients with negative MRD at HSCT (${{{\mathrm{ELN2022}}}}_{{{{\mathrm{MRD - adjusted}}}}}^{{{{\mathrm{refined}}}}}$ favorable), intermediate outcomes were observed in ${{{\mathrm{ELN2022}}}}_{{{{\mathrm{at}}}}\;{{{\mathrm{diagnosis}}}}}^{{{{\mathrm{refined}}}}}$ favorable risk patients with positive MRD at HSCT as well as ${{{\mathrm{ELN2022}}}}_{{{{\mathrm{at}}}}\;{{{\mathrm{diagnosis}}}}}^{{{{\mathrm{refined}}}}}$ adverse risk patients with negative MRD at HSCT (${{{\mathrm{ELN2022}}}}_{{{{\mathrm{MRD - adjusted}}}}}^{{{{\mathrm{refined}}}}}$ intermediate), and the most adverse outcomes had ${{{\mathrm{ELN2022}}}}_{{{{\mathrm{at}}}}\;{{{\mathrm{diagnosis}}}}}^{{{{\mathrm{refined}}}}}$ intermediate and adverse risk patients with positive MRD at HSCT (${{{\mathrm{ELN2022}}}}_{{{{\mathrm{MRD - adjusted}}}}}^{{{{\mathrm{refined}}}}}$ adverse).

**Fig. 5:**
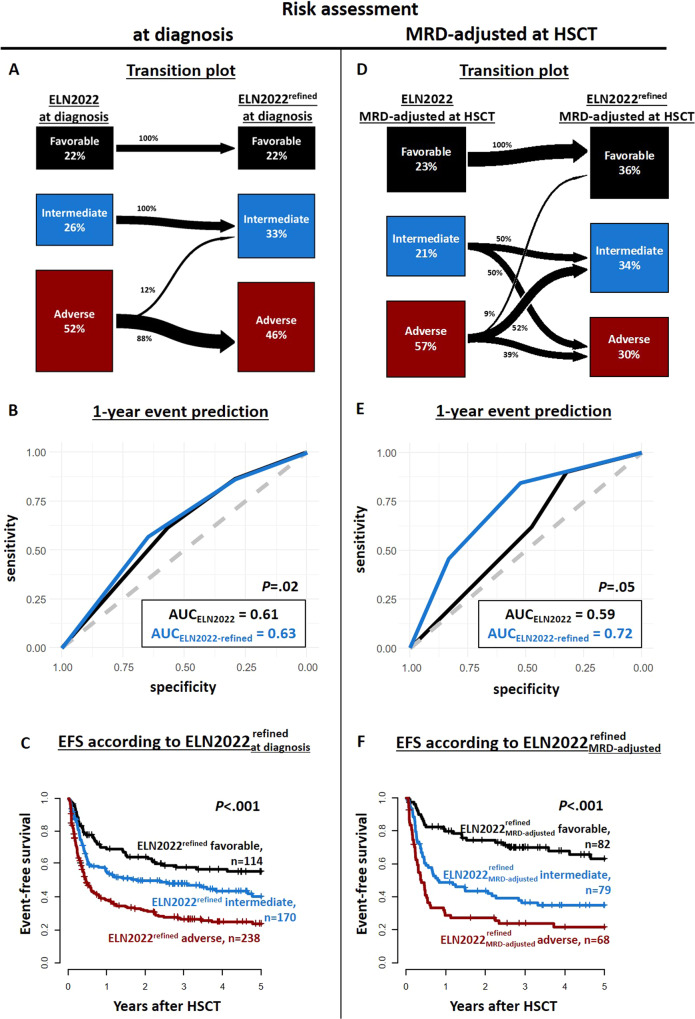
Outcomes according to the proposed refinement of the ELN2022 risk groups at diagnosis and MRD-adjusted at HSCT. **A** Transition plot for patients distribution from the ${{{\mathrm{ELN2022}}}}_{{{{\mathrm{at}}}}\;{{{\mathrm{diagnosis}}}}}$ risk to the ${{{\mathrm{ELN2022}}}}_{{{{\mathrm{at}}}}\;{{{\mathrm{diagnosis}}}}}^{{{{\mathrm{refined}}}}}$. **B** ROC curves comparison for suffering an event (relapse or death) within 1 year after HSCT between the ${{{\mathrm{ELN2022}}}}_{{{{\mathrm{at}}}}\;{{{\mathrm{diagnosis}}}}}$ and the ${{{\mathrm{ELN2022}}}}_{{{{\mathrm{at}}}}\;{{{\mathrm{diagnosis}}}}}^{{{{\mathrm{refined}}}}}$risk groups, **C** Event-free survival according to the three ${{{\mathrm{ELN2022}}}}_{{{{\mathrm{at}}}}\;{{{\mathrm{diagnosis}}}}}^{{{{\mathrm{refined}}}}}$ risk groups, **D** Transition plot for patients distribution from the ${{{\mathrm{ELN2022}}}}_{{{{\mathrm{MRD - adjusted}}}}}$ risk to the ${{{\mathrm{ELN2022}}}}_{{{{\mathrm{MRD - adjusted}}}}}^{{{{\mathrm{refined}}}}}$ risk at HSCT, **E** ROC curves comparison for suffering an event (relapse or death) within 1 year after HSCT between the ${{{\mathrm{ELN2022}}}}_{{{{\mathrm{MRD - adjusted}}}}}$ and the ${{{\mathrm{ELN2022}}}}_{{{{\mathrm{MRD - adjusted}}}}}^{{{{\mathrm{refined}}}}}$ risk groups at HSCT, and **F** Event-free survival according to the three ${{{\mathrm{ELN2022}}}}_{{{{\mathrm{MRD - adjusted}}}}}^{{{{\mathrm{refined}}}}}$risk groups at HSCT.

Comparing the c-statistics of the ELN2022 risk groups and our refined models at diagnosis as well as at HSCT (Fig. 5 and Supplementary Fig. S8), the refined models performed significantly better in predicting relapse (at diagnosis *P* = 0.007, at HSCT *P* = 0.001), or an event (at diagnosis *P* = 0.002, at HSCT *P* = 0.05) one year after HSCT, while only the refined model at diagnosis (*P* = 0.02), but not that at HSCT (*P* = 0.77) performed better in predicting death one year after HSCT. Definitions of the risk models are given in Supplementary Table S3).

## Discussion

With an increased understanding of AML biology, improved cytogenetic and molecular characterizations, and the availability of novel therapeutic compounds, adjustments to our prognostic guidance systems are inevitable. Very recently, this has been implemented by the updated ELN2022 risk classification system, which now—in addition to a conventional cytogenetic characterization—takes into account the mutation status of 13 genes—seven more than in the ELN classification of 2017 [2]. While numerous studies evaluated various included aberrations separately, to our knowledge no study validated the ELN2022 risk stratification in AML patients. Here we analyzed a cohort of 522 AML patients homogeneously treated with an allogeneic HSCT at our institution. Since this was a retrospective analysis, all patients were diagnosed with AML according to the WHO 2016 classification, and no patients belonged to the newly introduced category MDS/AML (comprising patients with >10% blasts at diagnosis).

Regarding the three ELN2022 risk groups at diagnosis, we observed distinct clinical and biological characteristics associated with ELN2022 adverse risk, including higher age, a higher amount of therapy-related or secondary AML, and different co-mutation profiles. While patients with ELN2022 adverse risk at diagnosis had a lower chance to achieve a CR/CRi prior to HSCT, intriguingly, the likelihood of an MRD-positive or MRD-negative CR/CRi at HSCT did not differ between the three ELN2022 risk groups (Table 1).

With respect to outcomes, the ELN2022 risk classification was able to allocate AML patients into three risk groups with significantly distinct outcomes, which was especially seen in patients younger than 60 years at HSCT, and in patients transplanted in first CR/CRi. However, in its most recent form the ELN2022 risk classification at diagnosis performed inferior in all analyzed endpoints compared to the ELN2017 risk classification (Supplementary Fig. S1).

When we analyzed the three ELN2022 risk groups separately, we observed no significantly different CIR, EFS, or OS between the distinct genetic aberrations characterizing favorable or intermediate ELN2022 risk (Fig. 3 and Supplementary Fig. S5). Noteworthy, patients with an *FLT3*-ITD AR higher or lower than 0.5 (as included in the ELN2017 risk classification) did not differ regarding their CIR (*P* = 0.13), EFS (*P* = 0.30) or OS (*P* = 0.30) after HSCT, supporting the removal of the *FLT3-*ITD AR from the ELN2022 risk stratification. While only a minority of our patient population received FLT3 inhibitors, and although subsequently restricted by patient numbers, outcomes tended to improve in ELN2022 intermediate risk in a subanalysis of AML patients treated in the era of new drugs or within the verum arm of a trial testing an FLT3 inhibitor (Supplementary Fig. S9). In contrast, the outcomes of patients allocated into the adverse ELN2022 risk group differed significantly, with the best outcomes in patients harboring mutations in the newly included genes *BCOR*, *EZH2*, *SF3B1*, *SRSF2*, *STAG2*, *U2AF1*, and *ZRSR2*, which now define adverse risk in the absence of a favorable risk aberration. Previous studies that showed a potential adverse prognostic impact of myelodysplasia-related gene mutations all included less than 50% of patients receiving a consolidating allogeneic HSCT [12, 24, 25]. Furthermore, two independent studies indicated that patients with myelodysplasia-related gene mutations might have improved outcomes with consolidating allogeneic HSCT, as compared to chemotherapy alone [12, 26]. Similarly, in our transplanted patient population, myelodysplasia-related gene mutations did not associate with adverse outcomes when no other adverse risk characteristics were present. One could speculate that an allogeneic HSCT might have the potential to overcome the adverse prognostic impact of myelodysplasia-related gene mutations. This is in line with our previous data indicating that patients with secondary AML only had adverse outcomes after HSCT when ELN2017 adverse-risk genetics are present [27], or that *SRSF2* mutations do not associate with adverse outcomes after allogeneic HSCT [28]. A recently published ASH abstract by Rausch et al. also indicated intermediate outcomes in patients characterized as adverse ELN2022 risk due to a myelodysplasia-related gene mutation in two independent cohorts treated within AMLCG or AML-SG study protocols [29]. Subsequently, whether myelodysplasia-related gene mutations really confer adverse outcomes in the context of the ELN2022 classification should be further evaluated. Some important additional diagnostic changes impacting complex karyotypes, *NPM1* and *TP53* were introduced by the ELN2022 regarding adverse risk, but affected only a few patients in our cohort (please see Supplementary Information).

Importantly, the ELN2022 now allows for adjustment of the assigned risk according to the MRD status during or after therapy: “a patient with favorable risk AML may be reclassified as intermediate risk or vice versa, based on the presence or absence of MRD, respectively” [3]. Following this suggestion, in our set, approximately half of patients with favorable or intermediate ${{{\mathrm{ELN2022}}}}_{{{{\mathrm{at}}}}\;{{{\mathrm{diagnosis}}}}}$ risk changed their risk at HSCT (Fig. 4A). This resulted in improved outcomes for MRD-adjusted favorable risk patients, while for MRD-adjusted intermediate risk patients, outcomes were comparable to those of with ${{{\mathrm{ELN2022}}}}_{{{{\mathrm{at}}}}\;{{{\mathrm{diagnosis}}}}}$ adverse risk.

With these findings, we sought to improve upon the current ELN2022 risk classification in the transplant context by introducing two changes. First, since the myelodysplasia-related gene mutations confer rather intermediate outcomes, we reclassified these patients as ${{{\mathrm{ELN2022}}}}_{{{{\mathrm{at}}}}\;{{{\mathrm{diagnosis}}}}}^{{{{\mathrm{refined}}}}}$ intermediate risk. This resulted in significantly better prediction of relapse, EFS, and OS (Fig. 5A–C and Supplementary Fig. S8A–D) 1 year after HSCT.

Next, we intended to improve upon the MRD adjustments. We previously demonstrated that the clinical value of the MRD status at HSCT is dependent on the ELN2017 risk at diagnosis. The higher the genetic risk, the more likely an “MRD-negative” patient relapses after HSCT and, thus, the lower the relative risk of relapse of “MRD-positive” patients in the same risk group [30]. While this remains true when the ELN2022 risk is considered, we still observed that the MRD status at HSCT has a strong prognosis-refining impact in patients within the ${{{\mathrm{ELN2022}}}}_{{{{\mathrm{at}}}}\;{{{\mathrm{diagnosis}}}}}^{{{{\mathrm{refined}}}}}$ adverse risk group (Supplementary Fig. S10). Subsequently, a ${{{\mathrm{ELN2022}}}}_{{{{\mathrm{MRD - adjusted}}}}}^{{{{\mathrm{refined}}}}}$ risk classification, in which we also adjusted the risk within the ${{{\mathrm{ELN2022}}}}_{{{{\mathrm{at}}}}\;{{{\mathrm{diagnosis}}}}}^{{{{\mathrm{refined}}}}}$ adverse risk group performed superior in predicting CIR and EFS than the originally proposed ${{{\mathrm{ELN2022}}}}_{{{{\mathrm{MRD - adjusted}}}}}$ (Fig. 5D–F and Supplementary Fig. S7E, F).

Apart from the ELN risk classification system, a variety of prediction models have been developed to improve outcome prediction and inform treatment decisions in AML. These include models like the knowledge bank approach introduced by Gerstung et al. to predict remission and relapse rates, but also NRM [31]. The clinical relevance of this model has been validated [32–35] and shown to be superior to the ELN2017 risk prediction in patients consolidated with chemotherapy [33], but not with allogeneic HSCT [35]. In addition to this approach, the increased use of machine learning and artificial intelligence approaches in outcome prediction will likely further impact AML risk assessment in the future [36].

In conclusion, our study is the first to explore the prognostic significance of the ELN2022 risk groups in AML. While the ELN2022 allows a risk stratification in AML patients undergoing allogeneic HSCT, it did not perform superior to the ELN2017 classification in outcome prognostication. When we refined the ELN2022 classification system by redistributing patients with diagnostic myelodysplasia-related gene mutations to the intermediate group and expanding the MRD-based reclassification to the adverse risk group, we improved the discriminative power of the ELN2022 risk classification. Further studies are needed to confirm our results, especially regarding the proposed refinements of the ELN2022 risk stratification at diagnosis and concerning the impact of MRD.

## Supplementary information

Supplemental Material

## Data Availability

The datasets generated during and/or analyzed during the current study are available from the corresponding author on reasonable request.

## References

[1] Döhner H, Estey EH, Amadori S, Appelbaum FR, Büchner T, Burnett AK (2010). Diagnosis and management of acute myeloid leukemia in adults: Recommendations from an international expert panel, on behalf of the European LeukemiaNet. Blood.

[2] Döhner H, Estey E, Grimwade D, Amadori S, Appelbaum FR, Ebert BL (2017). Diagnosis and management of AML in adults: 2017 ELN recommendations from an international expert panel. Blood.

[3] Döhner H, Wei AH, Appelbaum FR, Craddock C, DiNardo CD, Dombret H, et al. Diagnosis and management of AML in Adults: 2022 ELN Recommendations from an International Expert Panel. Blood. 2022; 140:1345–77. 10.1182/blood.2022016867 35797463

[4] Taube F, Georgi JA, Kramer M, Stasik S, Middeke JM, Röllig C (2022). CEBPA mutations in 4708 patients with acute myeloid leukemia: differential impact of bZIP and TAD mutations on outcome. Blood.

[5] Tarlock K, Lamble AJ, Wang Y-C, Gerbing RB, Ries RE, Loken MR (2021). CEBPA-bZip mutations are associated with favorable prognosis in de novo AML: a report from the Children’s Oncology Group. Blood.

[6] Wakita S, Sakaguchi M, Oh I, Kako S, Toya T, Najima Y (2022). Prognostic impact of CEBPA bZIP domain mutation in acute myeloid leukemia. Blood Adv.

[7] Bill M, Jentzsch M, Grimm J, Schubert K, Lange T, Cross M (2017). Prognostic impact of the European LeukemiaNet standardized reporting system in older AML patients receiving stem cell transplantation after non-myeloablative conditioning. Bone Marrow Transpl.

[8] Mroźek K, Marcucci G, Nicolet D, Maharry KS, Becker H, Whitman SP (2012). Prognostic significance of the European LeukemiaNet standardized system for reporting cytogenetic and molecular alterations in adults with acute myeloid leukemia. J Clin Oncol.

[9] Grimm J, Jentzsch M, Bill M, Goldmann K, Schulz J, Niederwieser D (2020). Prognostic impact of the ELN2017 risk classi fi cation in patients with AML receiving allogeneic transplantation. Blood Adv.

[10] Herold T, Rothenberg-Thurley M, Grunwald VV, Janke H, Goerlich D, Sauerland MC (2020). Validation and refinement of the revised 2017 European LeukemiaNet genetic risk stratification of acute myeloid leukemia. Leukemia.

[11] Boddu PC, Kadia TM, Garcia-Manero G, Cortes J, Alfayez M, Borthakur G (2019). Validation of the 2017 European LeukemiaNet classification for acute myeloid leukemia with NPM1 and FLT3-internal tandem duplication genotypes. Cancer.

[12] Gardin C, Pautas C, Fournier E, Itzykson R, Lemasle E, Bourhis JH (2020). Added prognostic value of secondary AML-like gene mutations in ELN intermediate-risk older AML: ALFA-1200 study results. Blood Adv.

[13] Pfrepper C, Klink A, Behre G, Schenk T, Franke G-N, Jentzsch M (2016). Risk factors for outcome in refractory acute myeloid leukemia patients treated with a combination of fludarabine, cytarabine, and amsacrine followed by a reduced-intensity conditioning and allogeneic stem cell transplantation. J Cancer Res Clin Oncol.

[14] Bryant A, Nivison-Smith I, Pillai ES, Kennedy G, Kalff A, Ritchie D (2014). Fludarabine Melphalan reduced-intensity conditioning allotransplanation provides similar disease control in lymphoid and myeloid malignancies: analysis of 344 patients. Bone Marrow Transpl.

[15] Kröger N, Iacobelli S, Franke GN, Platzbecker U, Uddin R, Hübel K (2017). Dose-reduced versus standard conditioning followed by allogeneic stem-cell transplantation for patients with myelodysplastic syndrome: a prospective randomized phase III study of the EBMT (RICMAC Trial). J Clin Oncol.

[16] Jentzsch M, Bill M, Grimm J, Schulz J, Schuhmann L, Brauer D (2020). High expression of the stem cell marker GPR56 at diagnosis identifies acute myeloid leukemia patients at higher relapse risk after allogeneic stem cell transplantation with the CD34+/CD38- population. Haematologica.

[17] Grimm J, Bill M, Jentzsch M, Beinicke S, Häntschel J, Goldmann K, et al. Clinical impact of clonal hematopoiesis in acute myeloid leukemia patients receiving allogeneic transplantation. Bone Marrow Transplant. 2019;54:1189–97. 10.1038/s41409-018-0413-0 30504903

[18] Jentzsch M, Bill M, Grimm J, Schulz J, Goldmann K, Beinicke S (2017). High BAALC copy numbers in peripheral blood prior to allogeneic transplantation predict early relapse in acute myeloid leukemia patients. Oncotarget.

[19] Jentzsch M, Bill M, Grimm J, Schulz J, Beinicke S, Häntschel J (2019). Prognostic impact of blood MN1 copy numbers before allogeneic stem cell transplantation in patients with acute myeloid leukemia. HemaSphere.

[20] Bill M, Grimm J, Jentzsch M, Kloss L, Goldmann K, Schulz J (2018). Digital droplet PCR-based absolute quantification of pre-transplant NPM1 mutation burden predicts relapse in acute myeloid leukemia patients. Ann Hematol.

[21] Lange T, Hubmann M, Burkhardt R, Franke GN, Cross M, Scholz M (2011). Monitoring of WT1 expression in PB and CD34 donor chimerism of BM predicts early relapse in AML and MDS patients after hematopoietic cell transplantation with reduced-intensity conditioning. Leukemia.

[22] Gray RJ (1988). A class of K-sample tests for comparing the cumulative incidence of a competing risk. Ann Stat.

[23] R Development Core Team. R: a language and environment for statistical computing. Vienna, Austria. 2017. http://www.R-project.org.

[24] Lindsley RC, Mar BG, Mazzola E, Grauman PV, Shareef S, Allen SL (2015). Acute myeloid leukemia ontogeny is defined by distinct somatic mutations. Blood.

[25] Van Der Werf I, Wojtuszkiewicz A, Meggendorfer M, Hutter S, Baer C, Heymans M (2021). Splicing factor gene mutations in acute myeloid leukemia offer additive value if incorporated in current risk classification. Blood Adv.

[26] Song G, Kim T, Ahn S, Jung S, Kim M, Yang D, et al. Allogeneic hematopoietic cell transplantation can overcome the adverse prognosis indicated by secondary-type mutations in de novo acute myeloid leukemia. Bone Marrow Transplant. 2022. 10.1038/s41409-022-01817-0. 10.1038/s41409-022-01817-0 36151367

[27] Jentzsch M, Grimm J, Bill M, Brauer D, Backhaus D, Goldmann K (2021). ELN risk stratification and outcomes in secondary and therapy-related AML patients consolidated with allogeneic stem cell transplantation. Bone Marrow Transplant.

[28] Grimm J, Jentzsch M, Bill M, Backhaus D, Brauer D, Küpper J (2021). Clinical implications of SRSF2 mutations in AML patients undergoing allogeneic stem cell transplantation. Am J Hematol.

[29] Rausch C, Rothenberg-Thurley M, Dufour AM, Schneider S, Gittinger H, Sauerland MC, et al. Validation of the 2022 European Leukemianet genetic risk stratification of acute myeloid leukemia. Blood Adv. 2022;6:1193–1206. 10.1038/s41375-023-01884-2 PMC10244159 37041198

[30] Jentzsch M, Grimm J, Bill M, Brauer D, Backhaus D, Pointner R (2021). Clinical value of the measurable residual disease status within the ELN2017 risk groups in AML patients undergoing allogeneic stem cell transplantation. Am J Hematol.

[31] Gerstung M, Papaemmanuil E, Martincorena I, Bullinger L, Gaidzik VI, Paschka P (2017). Precision oncology for acute myeloid leukemia using a knowledge bank approach. Nat Genet.

[32] Fenwarth L, Thomas X, de Botton S, Duployez N, Bourhis JH, Lesieur A (2021). A personalized approach to guide allogeneic stem cell transplantation in younger adults with acute myeloid leukemia. Blood.

[33] Bill M, Mrózek K, Giacopelli B, Kohlschmidt J, Nicolet D, Papaioannou D (2021). Precision oncology in AML: validation of the prognostic value of the knowledge bank approach and suggestions for improvement. J Hematol Oncol.

[34] Huet S, Paubelle E, Lours C, Grange B, Courtois L, Chabane K (2018). Validation of the prognostic value of the knowledge bank approach to determine AML prognosis in real life. Blood.

[35] Herrmann L, Bischof L, Backhaus D, Brauer D, Franke GN, Vucinic V (2022). Outcome prediction by the knowledge bank approach in acute myeloid leukemia patients undergoing allogeneic stem cell transplantation. Am J Hematol.

[36] Eckardt J-N, Röllig C, Metzeler K, Kramer M, Stasik S, Georgi J-A, et al. Prediction of complete remission and survival in acute myeloid leukemia using supervised machine learning. Haematologica. 2022. 10.3324/haematol.2021.280027. 10.3324/haematol.2021.280027 PMC9973482 35708137

