# Supplementary material for: Prognostic impact of the AML ELN2022 risk classification in patients undergoing allogeneic stem cell transplantation
Source: Blood Cancer J. 2022 Dec 19;12(12):170. doi: 10.1038/s41408-022-00764-9 (PMC9760726; doi:10.1038/s41408-022-00764-9)
Supplement: Supplementary file 1 — Supplemental Material [file 41408_2022_764_MOESM1_ESM.docx]

**Supplementary Material to**

**Prognostic Impact of the AML ELN2022 Risk Classification in Patients undergoing Allogeneic Stem Cell Transplantation**

*Jentzsch et al.*

**Chemotherapy protocols**

The majority of AML patients received standard Cytarabine-based induction protocols, *i.e.* with conventional 7+3 (n=107), conventional 7+3 with Midostaurin (n=9), CPX-351 (n=29)^1^ or sequential Azacytidine and OSHO induction (n=26); were treated within or according to the OSHO studies (#061 or #069,^2,3^ under or over 60 years, n=302), the Ratify Trial (n=7),^4^ the Unify Trial (ClinicalTrials.gov Identifier: NCT03512197, n=6) or the Quantum first trial (NCT02668653, n=8) and two patients were diagnosed with AML as children and treated within the AML BFM-2014 study. Eight and five patients received Azacytidine alone or Azacytidine and Venetoclax,^5^ respectively.

**Allogeneic HSCT**

Non-myeloablative (NMA) conditioning consisted of 3x30 mg/m^2^ Fludarabine and 2 Gy total body irradiation (TBI). Myeloablative conditioning (MAC) consisting of either 2x60 mg/kg body weight Cyclophosphamide and 12 Gy TBI or 5x30 mg/m^2^ Fludarabine and 8 Gy TBI. Reduced intensity conditioning (RIC) consisted of either busulfan (8 mg/kg orally or 6.4 mg/kg intravenously) or Treosulfan (3x10 g/m^2^) and 5x30 mg/m^2^ Fludarabine,^6^ Fludarabine and Melphalan,^7^ Fludarabine, Thiothepa, and Melphalan,^8^ or FLAMSA-based conditioning.^9^

**Immunosuppression and graft-versus-host disease**

For prevention of graft-versus-host disease (GvHD), all patients received an intravenous starting dose of 5 mg/kg body weight Cyclosporine A in two daily doses from day -1 which was adjusted to a whole-blood target level of 120-150 ng/ml for patients receiving FLAMSA conditioning or 200 ng/ml for all others.

Patients undergoing NMA-HSCT additionally received Mycophenolate Mofetil 3 g per day in three daily doses in case of unrelated HSCT or 2 g per day in two daily doses in case of related HSCT. None of the patients undergoing NMA-HSCT received *in vivo* T-cell depletion.

Patients receiving FLAMSA conditioning additionally received 2 g Mycophenolate Mofetil per day, which was stopped at day 28. Patients transplanted after RIC and MAC additionally received Methotrexate 15 mg intravenously on days +1, +3, +6, and +11 after HSCT, and RIC and MAC patients transplanted from an unrelated donor additionally received *in vivo* T-cell depletion with Thymoglobulin 2 mg/kg per day for three days. Cyclosporine A was reduced starting on day +42 and stopped on day 120 following FLAMSA conditioning and for all others reduced starting on day +84 or day +180 following related or unrelated HSCT, respectively. After NMA conditioning, Mycophenolate Mofetil was stopped at day +28 following related HSCT and tapered from days +40 to +96 following unrelated HSCT.^6^ Patients were evaluated for incidence of acute GvHD and chronic GvHD using established criteria of the Glucksberg grading system.^10^ Immunosuppression was prolonged or extended with systemic steroids in cases of GvHD (grade > 2 according to Glucksberg grading system).^10^ Requirement for acute GvHD was engraftment while requirement for chronic GvHD was engraftment and survival for at least 100 days after HSCT.

In patients receiving FLAMSA conditioning who did not develop signs of a GvHD, up to three prophylactic donor lymphocyte infusions (DLI) were administered per protocol after day 120 in increasing doses (5 x 10^5^/kg, 5 x 10^6^/kg, and 1 x 10^7^/kg recipient weight for patients receiving unrelated HSCT and 1 x 10^6^/kg, 5 x 10^6^/kg, and 1 x 10^7^/kg recipient weight for patients receiving related HSCT).

**Definition of complete remission and active disease**

Complete remission (CR) was defined as the presence of <5% blasts in bone marrow, neutrophils >1.0 x 10^9^/L, platelets >100 x 10^9^/L, absence of blasts with Auer rods in peripheral blood, independence of blood transfusion and no extramedullary disease.^11^ CR with incomplete peripheral recovery (CRi) was defined as CR with platelets <100 x 10^9^/L or neutrophils <1.0 x 10^9^/L. In patients receiving allogeneic HSCT, the presence of CR or CRi was confirmed within 28 days prior to HSCT by bone marrow and peripheral blood analysis. Active disease at HSCT was defined by a persisting blast count >5% in bone marrow, persisting blasts in peripheral blood or the detection of extramedullary disease.

**Multivariate analyses**

Multivariable proportional hazard models were constructed for CIR, EFS, and OS to evaluate the prognostic impact of the ELN2022 risk classification in AML patients by backward adjusting for other variables. The following variables were considered for multivariable analyses: sex, disease origin (*de novo* *vs* secondary), ELN2022 risk, age at HSCT (≥60 *vs* <60 years), disease status at HSCT (MRD^neg^ *vs* MRD^pos^), the number of remission at HSCT (first *vs* second *vs* no remission), the HCT-CI risk score (0 *vs* 1/2 *vs* 3 or more points), cytomegalovirus (CMV) status of recipient and donor (high-risk [+/-] *vs* all others), donor type (matched related *vs* matched unrelated *vs* mismatched unrelated), sex of the donor (female into male *vs* all others), and conditioning intensity (RIC/NMA *vs* MAC). Of these, variables significant at α=.10 in univariable analyses were considered for multivariable analyses. For all endpoints, hazard ratios with their corresponding 95% confidence intervals are indicated for every significant prognostic factor of the final model.

**Diagnostic qualifiers**

The ELN2022 introduced the diagnostic qualifiers „therapy-related“, „progressed from MDS or MDS/MPN“, and “germline predisposition”, which is appended to the specific AML diagnosis. While we did not include patients with a known germline predisposition into our patient set, we observed similar outcomes for the other two categories of diagnostic qualifiers, which had comparable CIR, but worse EFS and OS than patients without these clinical backgrounds (Supplementary Figure S5). Noteworthy, patients with a prior history of a myeloproliferative neoplasm (MPN), although a category not included as a diagnostic qualifier, also showed comparable outcomes to patients progressed from MDS or MDS/MPN and patients with therapy-related disease.

**Additional changes in the ELN2022 adverse risk group**

First, patients with a *NPM1* mutation, but coexisting adverse cytogenetics are now categorized to have adverse risk. In our cohort, this affected three patients, one with a concomitant complex karyotype was alive and relapse-free 4.6 years after HSCT. Two patients with concomitant complex and one with a monosomal karyotype relapsed within 28 days and 2.2 years after HSCT, respectively.

Second, a 10% VAF threshold for *TP53* mutations was defined to identify adverse risk. In our set, only 4 patients had a VAF below 10%. Two additionally had a complex karyotype and one a concomitant *STAG2* mutation, which still allocated them into the adverse ELN2022 risk group. These patients were alive and in morphologic remission 0.5, 1.2, and 6.2 years after HSCT, respectively. One patient was classified as having intermediate ELN2022 risk and relapsed 3.8 years after HSCT.

Finally, multiple trisomies are not regarded as complex karyotype in the ELN2022 risk classification. In our set, this affected 4 patients, 2 remained adverse risk due to a monosomy 7 and an *U2AF1* mutation, who relapsed 8.2 years after HSCT and died from NRM 106 days after HSCT, respectively. One patient was *NPM1* mutated and reclassified as ELN2022 favorable risk, and one patient had a *FLT3*-ITD and was reclassified as ELN2022 intermediate risk, both are alive and in remission 149 and 142 days after HSCT.

**Distinct immunophenotypes within to the ELN2022 risk groups**

The immunophenotype at diagnosis significantly differed between the three ELN2022 risk groups (Supplementary Table 2). With increasing ELN2022 risk, the bone marrow expression of immature antigens (*i.e.* CD34, and the leukemic stem cell population CD34+/CD38-), T-cellular differentiation antigens (*i.e.* CD2, and CD7) and the erythroid differentiation antigen Glycophorin A was higher. In contrast, antigens associated with myeloid and/or neutrophil differentiation (*i.e.* CD15, CD64, and CD33) and the pan-leukocyte antigen CD45 was significantly lower with increasing ELN2022 risk.

**Prognostic significance of the MRD status at HSCT within the ELN2022 risk groups**

We recently showed that the clinical value of the MRD status at HSCT also depends on the genetic risk at diagnosis assessed according to the ELN2017 risk groups.^12^ While the relapse risk in MRD positive patients was high, irrespective of the ELN2017 risk, MRD negative patients at HSCT only had a low relapse risk when they had a favorable or intermediate risk at diagnosis. In contrast, ELN2017 adverse risk patients with a positive MRD at HSCT still had relatively high relapse risk. We now also analyzed the clinical value of the MRD status in the context of the ELN2022 risk. Here, we observed a clinical value of MRD analyses in all ELN2022 risk groups, but again relatively high relapse probability in MRD negative AML patients with ELN2022 adverse risk at diagnosis (Supplementary Figure S10). The area under the curve for relapse prediction decreased from 0.78 in ELN2022 favorable to 0.73 in ELN2022 intermediate to 0.64 in ELN2022 adverse risk patients.

**References**

1. Lancet JE, Uy GL, Cortes JE, et al. Cpx-351 (cytarabine and daunorubicin) liposome for injection versus conventional cytarabine plus daunorubicin in older patients with newly diagnosed secondary acute myeloid leukemia. *J. Clin. Oncol.* 2018;36(26):2684–2692.

2. Büchner T, Schlenk RF, Schaich M, et al. Acute Myeloid Leukemia (AML): Different treatment strategies versus a common standard arm - Combined prospective analysis by the German AML Intergroup. *J. Clin. Oncol.* 2012;30(29):3604–3610.

3. Niederwieser D, Hoffmann VS, Pfirrmann M, et al. Comparison of Treatment Strategies in Patients over 60 Years with AML: Final Analysis of a Prospective Randomized German AML Intergroup Study. [abstract]. *Blood*. 2016;128(22):1066.

4. Stone RM, Mandrekar SJ, Sanford BL, et al. Midostaurin plus chemotherapy for acute myeloid leukemia with a FLT3 Mutation. *N. Engl. J. Med.* 2017;377(5):454–464.

5. DiNardo CD, Jonas BA, Pullarkat V, et al. Azacitidine and Venetoclax in Previously Untreated Acute Myeloid Leukemia. *N. Engl. J. Med.* 2020;383:617–629.

6. Kröger N, Iacobelli S, Franke GN, et al. Dose-reduced versus standard conditioning followed by allogeneic stem-cell transplantation for patients with myelodysplastic syndrome: A prospective randomized phase III study of the EBMT (RICMAC Trial). *J. Clin. Oncol.* 2017;35(19):2157–2164.

7. Bryant A, Nivison-Smith I, Pillai ES, et al. Fludarabine Melphalan reduced-intensity conditioning allotransplanation provides similar disease control in lymphoid and myeloid malignancies: Analysis of 344 patients. *Bone Marrow Transplant.* 2014;49(1):17–23.

8. Duque-Afonso J, Ihorst G, Waterhouse M, et al. Comparison of reduced-toxicity conditioning protocols using fludarabine, melphalan combined with thiotepa or carmustine in allogeneic hematopoietic cell transplantation. *Bone Marrow Transplant.* 2020;1–11.

9. Pfrepper C, Klink A, Behre G, et al. Risk factors for outcome in refractory acute myeloid leukemia patients treated with a combination of fludarabine, cytarabine, and amsacrine followed by a reduced-intensity conditioning and allogeneic stem cell transplantation. *J. Cancer Res. Clin. Oncol.* 2016;142(1):317–324.

10. Glucksberg H, Storb R, Fefer A, et al. Clinical manifestations of graft-versus-host disease in human recipients of marrow from HL-A-matched sibling donors. 1974;295–304.

11. Döhner H, Estey EH, Amadori S, et al. Diagnosis and management of acute myeloid leukemia in adults: Recommendations from an international expert panel, on behalf of the European LeukemiaNet. *Blood*. 2010;115(3):453–474.

12. Jentzsch M, Grimm J, Bill M, et al. Clinical value of the measurable residual disease status within the ELN2017 risk groups in AML patients undergoing allogeneic stem cell transplantation. *Am. J. Hematol.* 2021;96(7):E237–E239.

**Supplementary Tables**

**Supplementary Table 1:** Patients’ immunophenotype according to the ELN2022 genetic risk at diagnosis (n=522).

|  | **All patients**  **n=522** | **ELN2022 favorable**  **n=104** | **ELN2022 intermediate**  **n=137** | **ELN2022 adverse**  **n=271** | ***P*** |
| --- | --- | --- | --- | --- | --- |
| BM CD34 expression  median (range) | 20.8 (0-97) | 2.2 (0-84) | 9 (0-97) | 34 (0-93) | <.001 |
| BM CD38 expression  median (range) | 73 (1-98) | 83 (4-96) | 82 (1-98) | 63 (4-98) | <.001 |
| BM CD117 expression  median (range) | 35 (0-95) | 39 (1-91) | 43 (0-95) | 32 (1-91) | .12 |
| BM CD7 expression  median (range) | 15 (1-96) | 9 (1-91) | 14 (2-96) | 22 (2-90) | <.001 |
| BM CD56 expression  median (range) | 9 (0-97) | 6 (0-93) | 6 (1-97) | 11 (1-92) | .005 |
| BM Glykophorin A expression  median (range) | 10 (0-90) | 7 (1-90) | 6 (0-50) | 12 (1-73) | <.001 |
| BM CD2 expression  median (range) | 14 (1-97) | 10 (1-55) | 11 (2-97) | 20 (2-93) | <.001 |
| BM CD11b expression  median (range) | 15 (1-97) | 12 (1-93) | 15 (1-97) | 17 (1-91) | .63 |
| BM CD13 expression  median (range) | 58 (1-97) | 57 (1-94) | 70 (5-97) | 53 (1-96) | .006 |
| BM CD33 expression  median (range) | 67 (1-98) | 82 (2-98) | 83 (1-98) | 50 (3-96) | <.001 |
| BM CD15 expression  median (range) | 30 (1-94) | 53 (2-94) | 34 (1-90) | 22 (2-93) | <.001 |
| BM CD65 expression  median (range) | 19 (1-93) | 33 (1-90) | 25 (1-91) | 13 (1-93) | <.001 |
| BM CD14 expression  median (range) | 3 (1-74) | 2 (1-74) | 2 (1-56) | 3 (1-54) | .11 |
| BM CD64 expression  median (range) | 18 (0-98) | 26 (1-94) | 32 (0-98) | 11 (1-96) | <.001 |
| BM CD61 expression  median (range) | 4 (1-72) | 3 (1-72) | 3 (1-43) | 7 (1-67) | <.001 |
| BM CD45 expression  median (range) | 93 (29-100) | 94 (6-99) | 96 (53-100) | 89 (29-100) | <.001 |

*Abbreviations: BM, bone marrow; CD, cluster of differentiation*

**Supplementary Table 2:** Percent rates and 95% Confidence intervals for 3-year outcomes after HSCT of selected patient subgroups

|  | **CIR at 3 years** | **EFS at 3 years** | **OS at 3 years** |
| --- | --- | --- | --- |
|  | **% (95% CI)** | **% (95% CI)** | **% (95% CI)** |
| **All patients**  ELN2022 favorable  ELN2022 intermediate  ELN2022 adverse | 18 (8-21)  41 (33-50)  51 (44-57) | 58 (49-68)  47 (39-56)  30 (25-37) | 65 (56-75)  61 (53-71)  48 (42-55) |
| **ELN2022 adverse risk**  complex karyotype  other adverse risk cytogenetics  adverse-risk mutations (according to ELN2017)  myelodysplasia-related gene mutations (added in ELN2022) | 59 (48-68)  50 (39-60)  47 (27-64)  30 (14-47) | 17 (11-27)  32 (24-44)  42 (27-66)  55 (40-76) | 30 (21-42)  54 (44-66)  58 (41-81)  73 (59-91) |
| **MRD-adjusted outcomes**  ELN2022 favorable or intermediate, MRD-negative  ELN2022 favorable or intermediate, MRD-positive  ELN2022 adverse, transplanted in CR/CRi | 12 (5-20)  53 (40-64)  47 (44-57) | 69 (59-81)  33 (24-47)  37 (25-37) | 75 (66-86)  50 (39-64)  48 (42-55) |

**Supplementary Table 3:** Definition of the proposed risk models at diagnosis and at HSCT.

| **Group** | **Definition** |
| --- | --- |
| $\text{ELN2022}_{\text{at diagnosis}}$ | Three risk groups according to the genetic risk at diagnosis, as proposed by the ELN2022. |
| $\text{ELN2022}_{\text{MRD-adjusted}}$ | MRD adjustment only for patients with favorable or intermediate risk at diagnosis, as proposed by the ELN2022.  MRD-negative patients at HSCT with favorable or intermediate $\text{ELN2022}_{\text{at diagnosis}}$ risk defined the new favorable risk group, and MRD-positive patients at HSCT with favorable or intermediate $\text{ELN2022}_{\text{at diagnosis}}$ risk defined the new intermediate risk group. The adverse risk group remained defined by the genetic risk at diagnosis. |
| $\text{ELN2022}_{\text{at diagnosis}}^{\text{refined}}$ | $\text{ELN2022}_{\text{at diagnosis}}$ adverse risk patients harboring myelodysplasia-related gene mutations as sole adverse risk factor were re-classified as intermediate risk. The $\text{ELN2022}_{\text{at diagnosis}}$favorable risk group remained unchanged. |
| $\text{ELN2022}_{\text{MRD-adjusted}}^{\text{refined}}$ | All three $\text{ELN2022}_{\text{at diagnosis}}^{\text{refined}}$ risk groups were adjusted based on the MRD status at HSCT: MRD-adjusted $\text{ELN2022}_{\text{at diagnosis}}^{\text{refined}}$ favorable ($\text{ELN2022}_{\text{at diagnosis}}^{\text{refined}}$favorable and intermediate risk patients with negative MRD at HSCT), MRD-adjusted $\text{ELN2022}_{\text{at diagnosis}}^{\text{refined}}$intermediate ($\text{ELN2022}_{\text{at diagnosis}}^{\text{refined}}$ favorable risk patients with positive MRD at HSCT and $\text{ELN2022}_{\text{at diagnosis}}^{\text{refined}}$adverse risk patients with negative MRD at HSCT), MRD-adjusted $\text{ELN2022}_{\text{at diagnosis}}^{\text{refined}}$adverse ($\text{ELN2022}_{\text{at diagnosis}}^{\text{refined}}$ intermediate and adverse risk patients with positive MRD at HSCT). |

**Supplementary Figures**

**Supplementary Figure S1**

**Supplementary Figure S1. Ability of the ELN2017 (black curves) and ELN2022 (red curves) risk classification systems at diagnosis to predict outcomes in AML patients undergoing allogeneic HSCT.** Receiver Operator characteristics (ROC) curves for the prediction of **(A)** relapse, **(B)** relapse or death, and **(C)** death within one year after HSCT.

**Supplementary Figure S2**

**
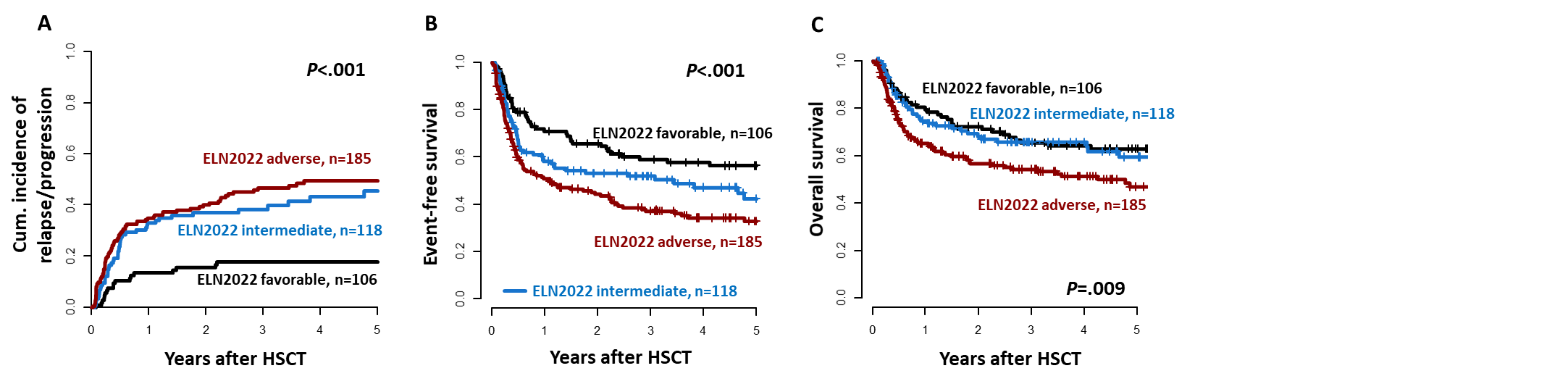
**

**Supplementary Figure S2. Outcomes according to the ELN2022 risk groups at diagnosis in AML patients transplanted in morphologic remission (n=408). (A)** Cumulative incidence of relapse/progression, **(B)** Event-free survival, and **(C)** Overall survival.

**Supplementary Figure S3**


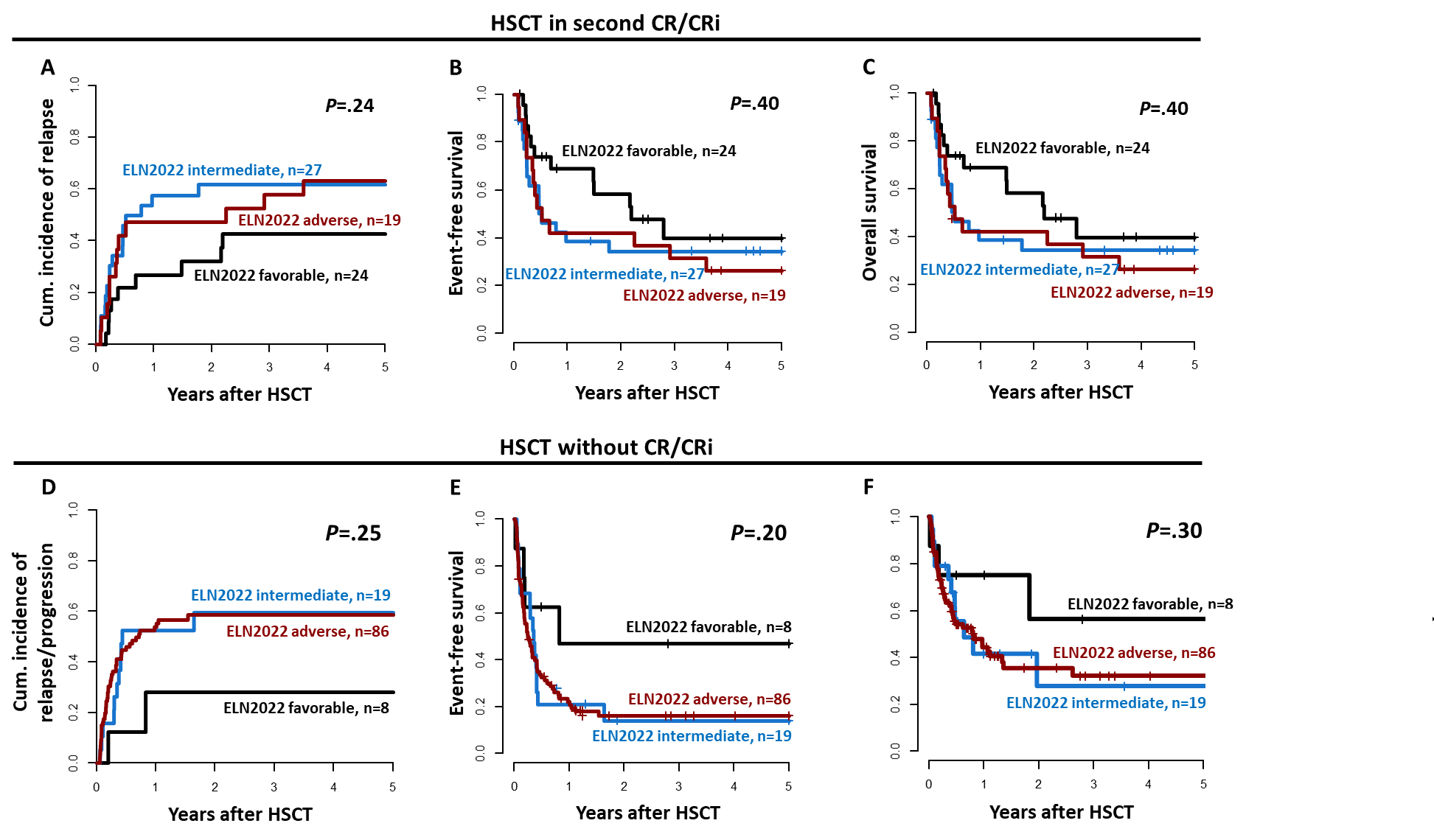


**Supplementary Figure S3. Outcomes according to the ELN2022 genetic risk groups at diagnosis within patients transplanted in first, second, or without morphologic remission. (A)** Cumulative incidence of relapse, **(B)** Event-free survival, and **(C)** Overall survival in AML patients transplanted in second remission (n=70). **(D)** Cumulative incidence of relapse/progression, **(E)** Event-free survival, and **(F)** Overall survival in AML patients transplanted without a remission (n=113).

**Supplementary Figure S4**


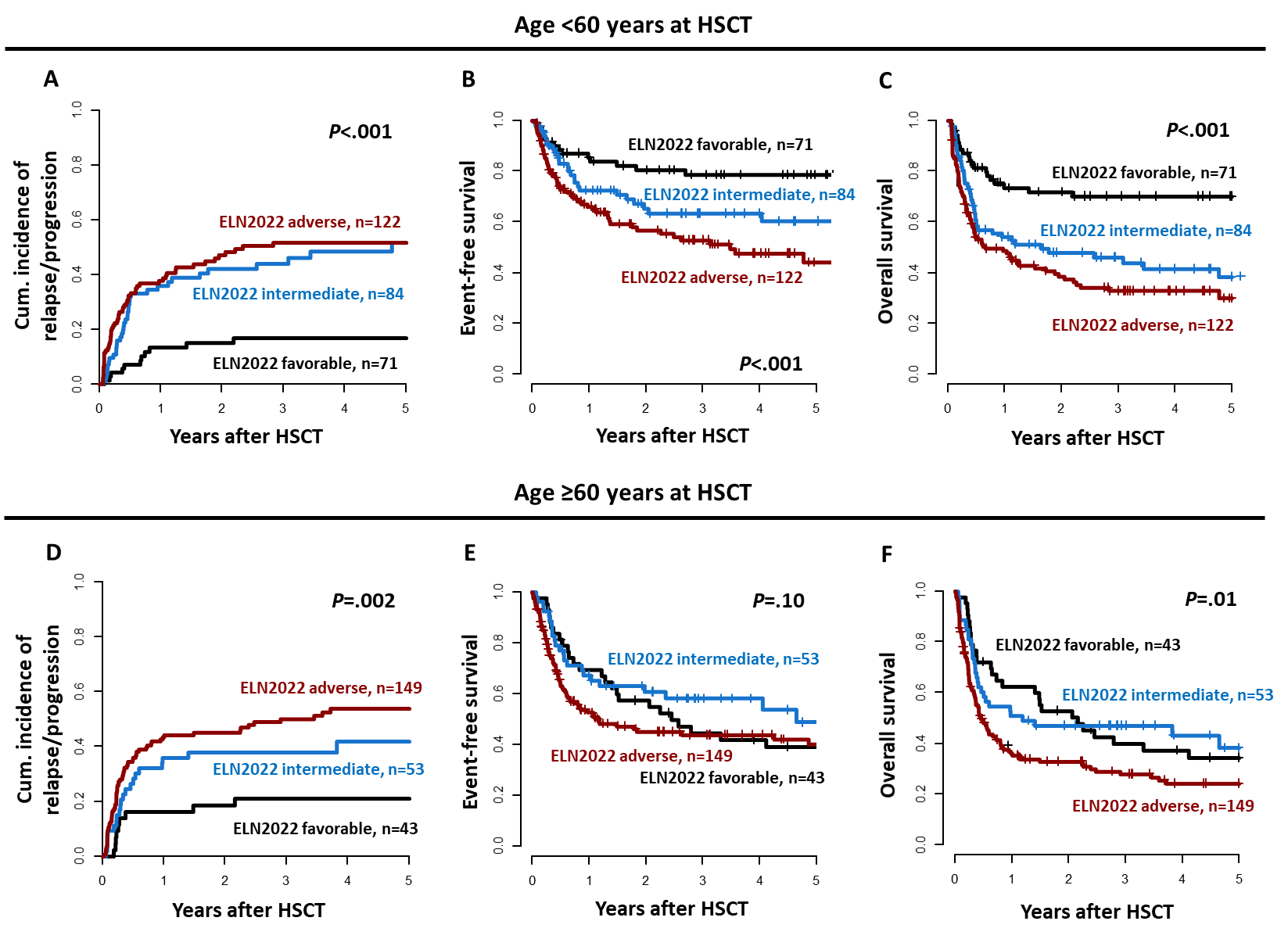


**Supplementary Figure S4. Outcomes according to the ELN2022 genetic risk groups at diagnosis within distinct age groups. (A)** Cumulative incidence of relapse/progression, **(B)** Event-free survival, and **(C)** Overall survival in AML patients younger than 60 years at HSCT (n=277). **(D)** Cumulative incidence of relapse/progression, **(E)** Event-free survival, and **(F)** Overall survival in AML patients 60 years and older at HSCT (n=245)

**Supplementary Figure S5**

**
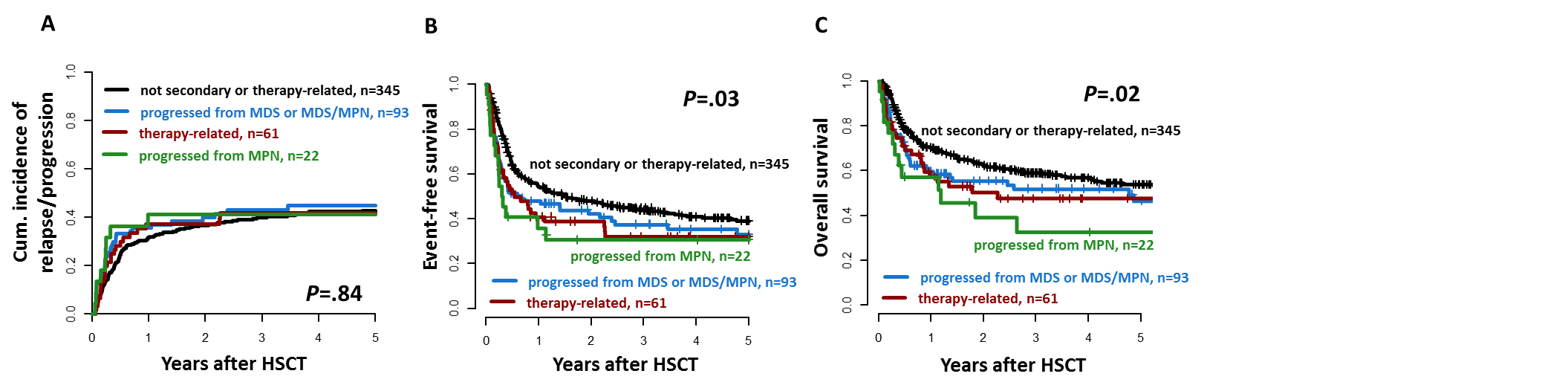
**

**Supplementary Figure S5. Outcomes according to the diagnostic qualifiers in the ELN2022 recommendations** (therapy-related *vs* progressed from MDS or MDS/MPN *vs* progressed from MPN [not mentioned as a diagnostic qualifier in the ELN2022] *vs* patients without these histories. **(A)** Cumulative incidence of relapse, **(B)** Event-free survival, and **(C)** Overall survival.

**Note: *P* values for subgroup comparisons**: therapy-related *vs* progressed from MDS or MDS/MPN, CIR *P*=.71, EFS *P*=.90, OS *P*=.90; no diagnostic qualifier *vs* therapy-related AML, CIR *P*=.76, EFS *P*=.05, OS *P*=.06; no diagnostic qualifier *vs* AML progressed from MDS or MDS/MPN, CIR *P*=.40, EFS *P*=.03, OS *P*=.04; no diagnostic qualifier *vs* AML progressed from MPN, CIR *P*=.67, EFS *P*=.06, OS *P*=.02.

**Supplementary Figure S6**


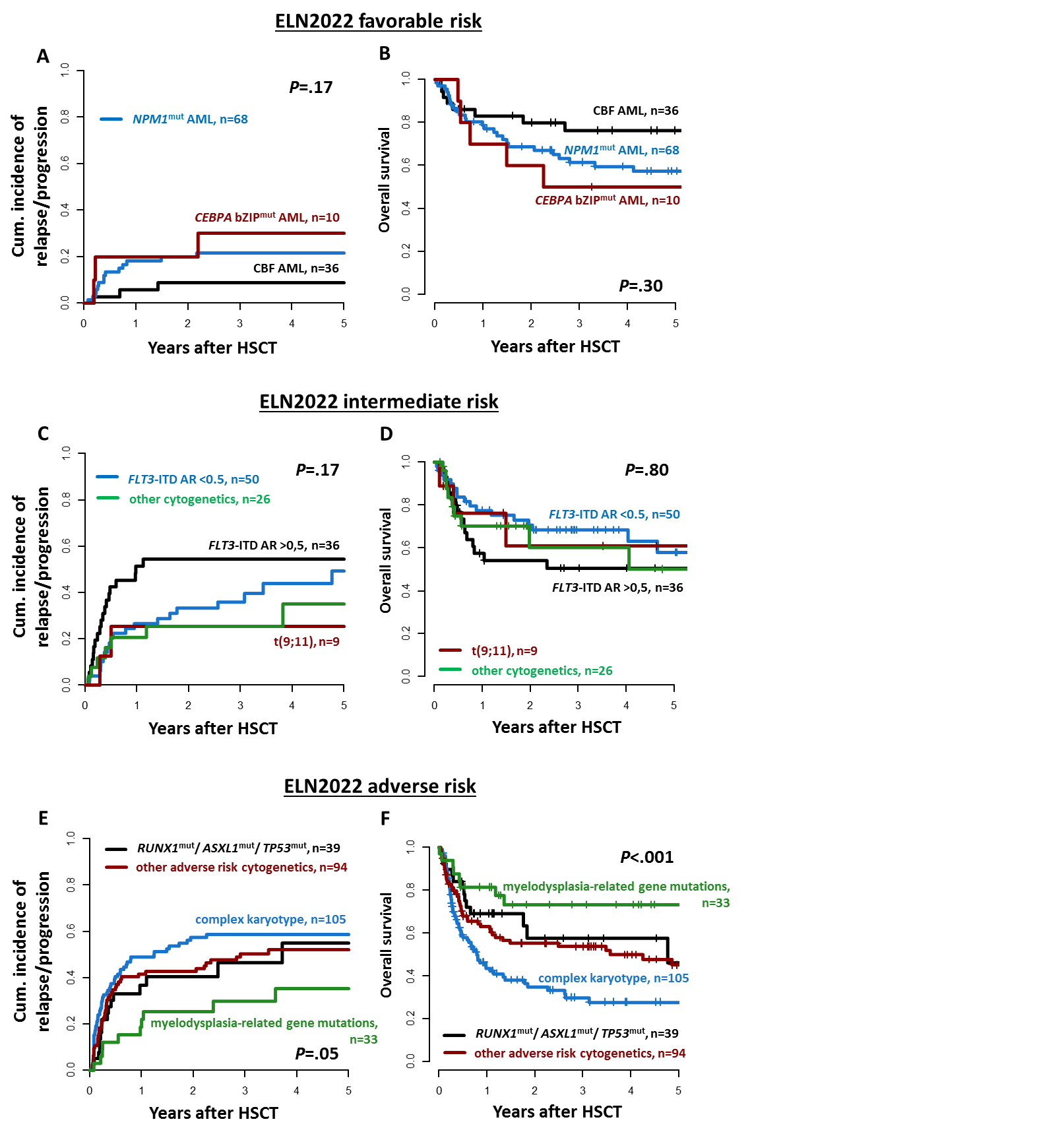


**Supplementary Figure S6. Outcomes within the three ELN2022 genetic risk groups at diagnosis. (A)** Cumulative incidence of relapse/progression, and **(B)** Overall survival in favorable ELN2022 risk AML patients (n=114). **(C)** Cumulative incidence of relapse/progression, and **(D)** Overall survival in intermediate ELN2022 risk AML patients (n=121). **(E)** Cumulative incidence of relapse/progression, and **(F)** Overall survival in adverse ELN2022 risk AML patients (n=271).

**Note: *P* values for subgroup comparisons: ELN2022 favorable risk:** CBF *vs NPM1*^mut^ CIR *P=*.20, OS *P*=.20, CBF *vs CEBPA* bZIP^mut^ CIR *P*=.09, OS *P*=.50, *NPM1*^mut^ *vs CEBPA* bZIP^mut^ *P*=.54; **ELN2022 intermediate risk:** *FLT3*-ITD AR >0.5 *vs* *FLT3*-ITD AR <0.5 CIR *P*=.13, OS *P*=.30, *FLT3*-ITD AR >0.5 *vs* t(9;11) CIR *P*=.14, OS *P*=.60, *FLT3*-ITD AR >0.5 *vs* other cytogenetics CIR *P*=.06, OS *P*=90, *FLT3*-ITD AR <0.5 *vs* t(9;11) CIR *P*=.42, OS *P*>.99, *FLT3*-ITD AR <0.5 *vs* other cytogenetics CIR *P*=.50, OS *P*=.59, t(9;11) *vs* other cytogenetics CIR *P*=.76, OS *P*=.70). **ELN2022 adverse risk:** ELN2017 defined high risk mutations *vs* myelodysplasia-related gene mutation CIR *P*=.11, *P*=.40, ELN2017 defined high risk mutations *vs* complex karyotype CIR *P*=.24, OS *P*=.01; ELN2017 defined high risk mutations *vs* other adverse risk karyotypes CIR *P*=.74, OS *P*=.40, myelodysplasia-related gene mutation *vs* complex karyotype CIR *P*=.006, OS *P*<.001, myelodysplasia-related gene mutation *vs* other adverse risk karyotypes CIR *P*=.05, OS *P*=.10, complex karyotype *vs* other adverse risk karyotypes CIR *P*=.25, OS *P*=.009.

**Supplementary Figure S7**

**Supplementary Figure S7. Outcomes according to the three ELN2022 risk groups at diagnosis and the MRD status at HSCT. (A)** Cumulative incidence of relapse/progression, MRD^pos^ *vs* MRD^neg^, ELN2022 favorable *P*<.001, ELN2022 intermediate *P*<.001, ELN2022 adverse *P*=.002;  **(B)** Event-free survival, MRD^pos^ *vs* MRD^neg^, ELN2022 favorable *P*=.001, ELN2022 intermediate *P*<.001, ELN2022 adverse *P*=.01; and **(C)** Overall survival, MRD^pos^ *vs* MRD^neg^, ELN2022 favorable *P*=.06, ELN2022 intermediate *P*=.01, ELN2022 adverse *P*=.50).

**Supplementary Figure S8**

**Supplementary Figure S8. Outcomes according to the proposed refined ELN2022 risk classification at diagnosis and at HSCT.** **(A)** ROC curves comparison for suffering relapse within 1 year after HSCT between the $\text{ELN2022}_{\text{at diagnosis}}$ and the $\text{ELN2022}_{\text{at diagnosis}}^{\text{refined}}$ risk groups, **(B)** Cumulative incidence of relapse/progression according to the three $\text{ELN2022}_{\text{at diagnosis}}^{\text{refined}}$ risk groups, **(C)** ROC curves comparison for suffering death within 1 year after HSCT between the ELN2022_at diagnosis_ and the $\text{ELN2022}_{\text{at diagnosis}}^{\text{refined}}$ risk groups, and **(D)** Overall survival according to the three $\text{ELN2022}_{\text{at diagnosis}}^{\text{refined}}$ risk groups. **(E)** ROC curves comparison for suffering relapse within 1 year after HSCT between the ELN2022 and the $\text{ELN2022}_{\text{MRD-adjusted}}^{\text{refined}}$ risk groups at HSCT, **(F)** Cumulative incidence of relapse according to the three $\text{ELN2022}_{\text{MRD-adjusted}}^{\text{refined}}$risk groups at HSCT, **(G)** ROC curves comparison for suffering death within 1 year after HSCT between the ELN2022 and the $\text{ELN2022}_{\text{MRD-adjusted}}^{\text{refined}}$risk groups at HSCT, and **(H)** Overall survival according to the three $\text{ELN2022}_{\text{MRD-adjusted}}^{\text{refined}}$ risk groups at HSCT.

**Supplementary Figure S9**

**
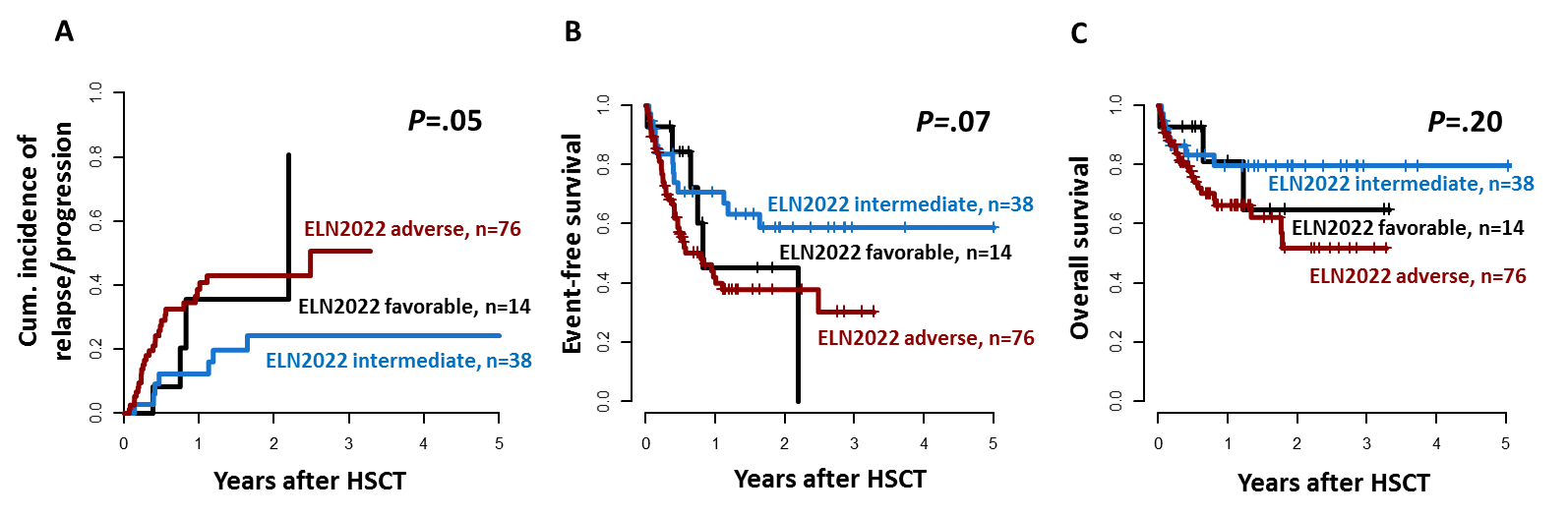
**

**Supplementary Figure S9. Outcomes according to the ELN2022 genetic risk groups at diagnosis in AML patients treated in the era of new drugs. (A)** Cumulative incidence of relapse/progression, **(B)** Event-free survival, and **(C)** Overall survival.

**Supplementary Figure S10**

**
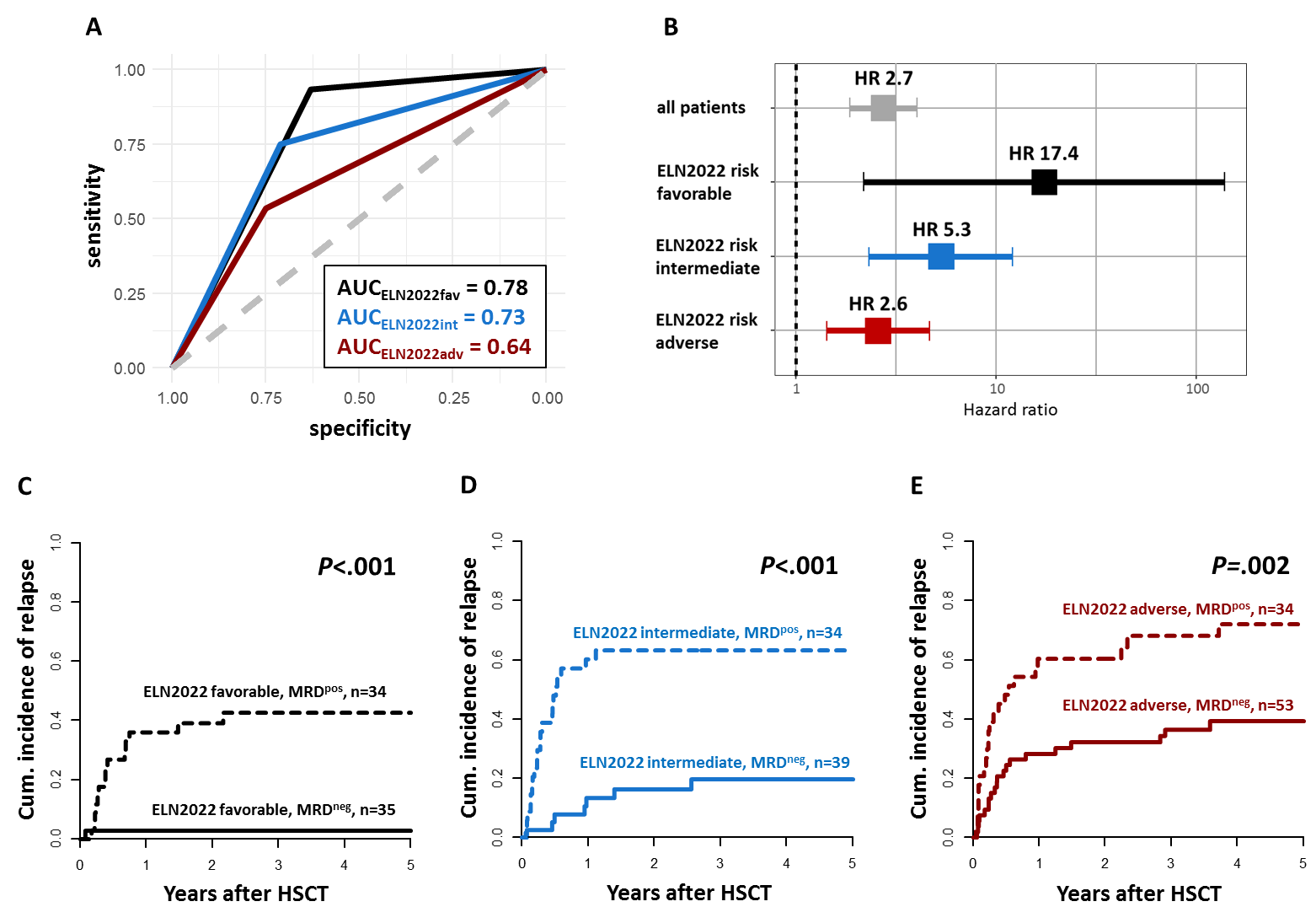
**

**Supplementary Figure S10. Relapse incidences according to the MRD status at HSCT within the three ELN2022 risk groups at diagnosis. (A) ROC comparison, (B)** Cumulative incidence of relapse in patients with favorable ELN2022 genetic risk at diagnosis according to MRD status at HSCT**, (C)** Cumulative incidence of relapse in patients with intermediate ELN2022 genetic risk at diagnosis according to MRD status at HSCT, and **(D)** Cumulative incidence of relapse in patients with favorable ELN2022 genetic risk at diagnosis according to MRD status at HSCT**.**
